# Evaluation of a tablet‐based assessment tool for measuring cognition among children 4–6 years of age in Ghana

**DOI:** 10.1002/brb3.2749

**Published:** 2022-09-09

**Authors:** Haiying Yuan, Maku Ocansey, Seth Adu‐Afarwuah, Margaret Sheridan, Amar Hamoudi, Harriet Okronipa, Sika M. Kumordzie, Brietta M. Oaks, Elizabeth Prado

**Affiliations:** ^1^ Institute for Global Nutrition, Department of Nutrition University of California Davis California; ^2^ Department of Nutrition and Food Science University of Ghana Accra Ghana; ^3^ Department of Psychology and Neuroscience University of North Carolina Chapel Hill North Carolina; ^4^ Sanford School of Public Policy Duke University Durham North Carolina; ^5^ Department of Nutrition and Food Sciences University of Rhode Island Kingstown Rhode Island

**Keywords:** cognition assessment, low‐ and middle‐income countries, tablet tasks

## Abstract

**Objectives:**

To investigate several basic psychometric properties, including construct, convergent and discriminant validity, of the tablet‐based Rapid Assessment of Cognitive and Emotional Regulation (RACER) among children aged 4–6 years in Ghana.

**Methods:**

We investigated whether RACER tasks administered to children in Ghana could successfully reproduce expected patterns of performance previously found in high‐income countries on similar tasks assessing inhibitory control (e.g., slower responses on inhibition trials), declarative memory (e.g., higher accuracy on previously seen items), and procedural memory (e.g., faster responses on sequence blocks). Next, we assessed the validity of declarative memory and inhibitory control scores by examining associations of these scores with corresponding paper‐based test scores and increasing child age. Lastly, we examined whether RACER was more sensitive than paper‐based tests to environmental risk factors common in low‐ and middle‐income countries (LMICs).

**Results:**

Of the 966 children enrolled, more than 96% completed the declarative memory and inhibitory control tasks; however, around 30% of children were excluded from data analysis on the procedural memory task due to missing more than half of trials. The performance of children in Ghana replicated previously documented patterns of performance. RACER inhibitory control accuracy score was significantly correlated with child age (*r* (929) = .09, *p* = .007). However, our findings did not support other hypotheses.

**Conclusions:**

The high task completion rates and replication of expected patterns support that certain RACER sub‐tasks are feasible for measuring child cognitive development in LMIC settings. However, this study did not provide evidence to support that RACER is a valid tool to capture meaningful individual differences among children aged 4–6 years in Ghana.

## Graphical abstract

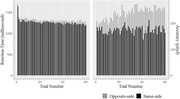

An estimated 250 million children under 5 years of age in low‐ and middle‐income countries (LMICs) are at risk of not fulfilling their developmental potential due to a variety of biological and psychosocial risk factors, such as childhood undernutrition, infectious diseases, exposure to toxins, and lack of cognitive stimulation and early learning opportunities (Black et al., 2017; Walker et al., 2007). The United Nations’ 193 members have adopted Sustainable Development Goal 4.2 to “ensure that all girls and boys have access to quality early childhood development, care, and preprimary education so that they are ready for primary education” (UN, 2015). Valid cognitive assessments for young children are needed to evaluate interventions and to inform evidence‐based policies and programs aiming to achieve this goal. However, well‐validated and reliable assessment tests for measuring young children's cognition in LMICs are limited (Semrud‐Clikeman et al., 2017).

Most cognitive assessments have been developed in high‐income countries (HICs) and may not show the same reliability and validity when translated to a different language and culture as compared to the validity in the original context (Greenfield, 1997). For example, the items in the test might not be relevant to the target culture; thus, they may not measure the underlying construct they were designed to measure. In addition to the need for cross‐cultural adaptation, other barriers to implementing cognitive assessments in many LMICs are multilingual contexts that require test administration in multiple languages and a lack of trained and certified personnel in cognitive assessment. Another barrier is that teachers and community health workers who might be tasked with administering such assessments already have a large existing workload.

The decreasing cost and increasing availability of tablets over the last decade has led to increased use of tablet‐based cognitive assessments, mainly in HICs. Using tablets allows researchers to create engaging, interactive, game‐like tests that are not only low‐cost but also quick to administer. Studies in HICs have reported that children from the age of two are capable of interacting with tablets, understanding game‐like cognitive tasks, and using tablets to make responses according to instructions (Frank et al., 2016; Semmelmann et al., 2016). Among children aged 6–14 years, such tablet‐based assessments have shown high test–retest reliability, internal reliability, convergent validity, and discriminant validity (Kanerva et al., 2019; Moore et al., 2017; Obradović et al., 2018; Pitchford & Outhwaite, 2016).

In contrast, few studies have reported evidence for the reliability and validity of tablet‐based tasks among children in LMIC settings (Bhavnani et al., 2019). In one such study, Bangirana et al. (2015) investigated the psychometric properties of a set of computer‐based cognitive tasks assessing visuomotor processing speed and working memory among Ugandan Children aged 5–13 years. They found low to moderate test–retest reliability (*r* = 0.35–0.57) and good convergent validity (*r* > 0.30) with other corresponding developmental tests and factors, such as age, education, and schooling. Recently, some researchers have initiated further efforts to develop tablet‐based assessment tools that may be appropriate for children and adolescents in LMICs, which includes Oxford Cognitive Screen Plus (Demeyere et al., 2021; Rowe et al., 2021), Developmental assessment on an E‐Platform (Bhavnani et al., 2019), Executive Function Touch (Willoughby et al., 2019), and the iPad version of National Institutes of Health Tool Box (Kabundula et al., 2022; Wray et al., 2020). The assessment tool investigated in this study, the Rapid Assessment of Cognitive and Emotional Regulation (RACER) is one of those efforts (Ford et al., 2019).

If the tablet‐based RACER tasks are valid in LMICs, they could provide several advantages over the paper‐and‐pencil‐based assessments that are widely used. The quick administration time (1–7 min per task) of RACER is important to minimize participant burden and facilitate assessments of large numbers of children who may be participants in a large trial or program evaluation. The use of tablet‐based cognitive assessments reduces the necessity for professional skills and intensive training of data collectors to administer the tests. Traditional paper‐based tests require a tester to interact with the child and to observe and record if the child accurately responds to each item administered. In contrast, tablet‐based tests are completed through participant‐tablet interaction, so a child's performance may be less influenced by the way the tester interacts with the child (Frank et al., 2016). Moreover, tablet‐based tasks can collect continuous and precise measures of response times and the touch locations of children's responses on a screen while maintaining a low burden on the test administrator. Although some paper‐based tasks can be timed using a method such as a stopwatch or an observer can record the location of a target that participants select, they cannot discriminate the precise differences in milliseconds or pixels as tablet‐based tasks are able to do. Response time reflects processing speed, which is driven by the speed of neuronal transmission (Chopra et al., 2018) and is closely related to performance on higher‐order cognitive tasks (i.e., reasoning and memory) throughout the lifespan (Kail & Salthouse, 1994). These precise scores on response times and touch locations may be more sensitive to children's individual differences in cognitive abilities than simple accuracy scores, such as the percent of total questions answered correctly, that we usually obtain from paper‐based assessments.

RACER was designed by Sheridan and her team, and it includes six short game‐like tasks to measure declarative memory, procedural memory, inhibitory control, and working memory. The RACER tasks were designed to be easily and quickly administered to and by those with no particular literacy or numeracy knowledge. All tasks use simple shapes. They are administrated on a touch‐screen tablet, and participants are instructed to respond by touching the shapes, which is an intuitive response. RACER requires limited tester training and verbal instructions. It automatically runs instructional videos, ensures that participants understand the tasks through passing practice trials prior to administration of each game, and automatically records responses. RACER can be administrated offline without any internet connection and on battery power; thus, the infrastructure requirement at the point of assessment is minimal (Ford et al., 2019; Kim et al., 2020). RACER has been shown to be easy to administer in widely variable and challenging assessment environments in LMICs, such as children aged 7–11 years in Lebanon and Niger (Ford et al., 2019), adolescents aged 12–18 years in Jordan (Chen et al., 2019), and primary school‐aged Syrian refugee children (Kim et al., 2020). These previous studies mainly focused on two RACER tasks, working memory and inhibitory control, and investigated school‐aged children. In this study, we examined three tasks (declarative memory, procedural memory, and inhibitory control) in a younger population, specifically children aged 4–6 years, and in a different LMIC setting, Ghana.

Assessment of declarative memory, procedural memory, and inhibitory control at this age, which is just before the typical age children start school in most countries, could be important for several reasons. Declarative memory and procedural memory are involved in many aspects of cognition, and they play critical roles in children's motor, social, and language development (Bjorklund, 2005; Tulving, 1972; Lieberman, 2000; Vakil et al., 2015; Ullman, 2001a, 2001b, 2004). Inhibitory control is another central concept in theories of child development (Carlson & Moses, 2001), and it has also been shown to predict academic and career success, socioemotional well‐being, and mental health (Anzman‐Frasca et al., 2015; Dong et al., 2012; Hennessy et al., 2019; Rhoades et al., 2009). Our previous study showed that nutritional intervention had a long‐term benefit for children's procedural memory, and poverty‐related malnutrition risk factors (e.g., maternal mid‐upper arm circumference, child height) were significantly associated with children's difficulties in executive function and declarative memory (Prado et al., 2017). The availability of valid and easy‐to‐use assessments at the age of school entry could be important to evaluate interventions conducted in early childhood and children's school readiness.

Our aims were, in a sample of children aged 4–6 years in Ghana, (1) to investigate whether three RACER tasks could successfully reproduce response patterns showing inhibitory control, and declarative and procedural learning, replicating patterns that have been found among children the same age in HICs; (2) to assess convergent validity and discriminant validity of declarative memory and inhibitory control scores, by examining associations of these RACER scores with (2a) paper‐based scores from tasks measuring declarative memory and inhibitory control and (2b) increasing child age; and (3) to examine whether the sensitivity of the RACER tasks to several risk factors for adverse developmental outcomes commonly experienced by children in LMICs will be similar to or higher than the sensitivity of the paper‐based tasks.

In accordance with previous findings in HICs (Jordan et al., 2007; Lum et al., 2010; Martin‐Rhee & Bialystok, 2008; Thomas & Nelson, 2001), we hypothesized that young children in Ghana would show a similar pattern of test performance as measured by the RACER tasks, compared to their peers in HICs tested using the same paradigms (Aim 1), and that the RACER tasks would show high validity (Aim 2a). We also hypothesized that the declarative memory and inhibitory control scores would show significant associations with age (Aim 2b), consistent with findings that these scores increase with age among children in HICs (Collier et al., 2001; DiGiulio et al., 1994; Kochanska et al., 1997; Macdonald et al., 2014; Reck & Hund, 2011). Research on the effects of age on the performance of children in procedural memory tasks has not drawn a consistent conclusion (Bauer, 2008; Collier et al., 2001; Lum et al., 2010; Zwart et al., 2019). Our fourth hypothesis (aim 3) was that the RACER scores would be significantly associated with psychosocial and biological risk factors commonly experienced by children in LMICs. Previous studies have shown that adverse developmental outcomes occur when young children are exposed to risk factors such as low maternal education (Carneiro et al., 2013; Noble et al., 2015), low household assets (Amso & Lynn, 2017), lack of learning opportunities and caregiving responsiveness (Melhuish et al., 2008; Rubio‐Codina et al., 2016), child growth stunting (Li et al., 2004), and iron‐deficiency anemia (Grantham‐McGregor & Ani, 2001; Lozoff et al., 2006). We hypothesized that RACER scores would be negatively correlated with these risk factors.

## 1 METHODS

### 1.1 Participants

This study investigated the psychometric properties of a tablet‐based assessment tool, RACER, in a large sample of young children aged 4–6 years in Ghana. Ghana is a sub‐Saharan country in Africa with a population of more than 30 million. An estimated 17.5% of children under age 5 years in Ghana are stunted, 7% wasted, 11% underweight, and 66% anemic (World Bank, 2022).

Participants were children of women who participated in the International Lipid‐Based Nutrient Supplements (iLiNS) DYAD (indicating the mother–child dyad) trial in Ghana from 2009 to 2014 (*n* = 1320) (Adu‐Afarwuah et al., 2016). In 2016, all parents or caregivers were contacted for enrollment in a follow‐up study, and written informed consent was obtained. Ethical approval for this study was obtained from the ethics committees of the University of California, Davis, the University of Ghana, and the Ghana Health Service.

### 1.2 Procedures

Detailed descriptions of the study procedures for the randomized trial and the first follow‐up study (Adu‐Afarwuah et al., 2016; Ocansey et al., 2019) have been published elsewhere, and information relevant to this study is summarized here. At enrollment into the original trial, maternal and household information, including maternal education and household assets, was collected by trained fieldworkers using a questionnaire. In 2016, field staff visited participants’ homes to explain the follow‐up study, obtain consent for participation, and collect updated sociodemographic information. Consenting participants were scheduled for a lab visit for assessment of hemoglobin and a visit to the test center for neurobehavioral, anthropometric, and other assessments. An additional home visit was conducted to administer the Home Observation for the Measurement of the Environment (HOME) inventory (Caldwell & Bradley, 2003) to measure responsive care and learning opportunities in the home environment. At the test center visit, neurobehavioral assessments, including both paper‐based tasks and the RACER tasks, were administered in a private testing room to minimize distractions. The data collection team consisted of five field workers recruited from the study area who were proficient in the local languages. One field worker had an undergraduate degree, and the other four finished high school. They were trained by a doctoral student researcher trained in child neurobehavioral assessments and an international developmental psychologist. They were all experienced with developmental assessments of children through previously administering the 18‐month developmental assessments for the iLiNS trial. The RACER tasks were administered on Dell Latitude 5175 tablets with a 10.8‐inch screen, using an android app programmed for the RACER tasks.

### 1.3 Adaptation of measures and quality control

The instructions for the RACER tasks and the two paper‐based tests were translated into the local languages in the study area. The language of administration of the tasks was the same as the child's primary language spoken at home. The tasks were then adapted to the local setting through an iterative process involving two pilot tests, each with 30 children age 4–6 years in the study area. Test materials and procedures were reviewed and modified after each pilot test (Ocansey et al., 2019). For the RACER tasks, children were given several practice items to assess whether they had understood the instructions. If the child did not pass the practice items, the instructions and practice items were repeated up to four times before starting the test until the child passed the practice items, demonstrating understanding of how to perform the task. All tasks worked well with children in the study area, the instructions were clearly understood, and the scores showed expected distributions for the target age. Therefore, no modifications were made to test materials or procedures.

During the data‐collection period, we conducted quarterly knowledge and practice‐based evaluations to ensure that the administration and ratings of the data collectors were standardized. We evaluated the interrater agreement at the beginning and during the last quarter of data collection. For each of the five trained data collectors, we video‐recorded one child testing session and one caregiver interview. All trained data collectors and their supervisors watched each video and independently scored the test or interview. For each data collector, the percentage of item scores that agreed with the supervisor was calculated.

### 1.4 Developmental assessment measures

The neurodevelopmental assessment battery administered at the test center visit included three tablet‐based RACER tasks assessing declarative memory, procedural memory, and inhibitory control, and two corresponding paper‐based tests for inhibitory control and declarative memory. To our knowledge, there is no paper‐based procedural memory task available.

#### 1.4.1 Inhibitory control tasks

The RACER inhibitory control task was based on the “Simon Task,” a cognitive experiment used widely to measure the inhibitory control abilities of young children in HICs (O'Leary & Barber, 1993; Simon & Rudell, 1967). There were two conditions in the task with a total of 30 trials per condition: same‐side and opposite‐side. In the same‐side trials, a yellow dot appeared on either the left side or the right side of the tablet screen, and children were instructed to touch the center of the dot as fast as possible. In the opposite‐side trials, a pink‐and‐black striped dot appeared, and the children were instructed to touch the center of the opposite side of the screen as fast as possible. The tablet recorded reaction time (RT) from stimuli appearance to child touch and the *x* and *y* coordinates of the location of each touch. Accuracy was calculated as the horizontal distance from the touch response to the target, defined as the difference in the *x*‐coordinate of the touch response from the *x*‐coordinate of the target location. Differences in performance between same‐side (baseline) trials and opposite‐side (challenge) trials reflected a child's inhibitory control ability. We expected that the participants would show the expected pattern of responding more quickly and accurately in the same‐side trials than in the opposite‐side trials because of failure to perfectly override the impulse to touch the presented dot. The test–retest reliabilities for RT and Accuracy were 0.35 and 0.86, respectively.

Numerous paper‐based tasks for measuring inhibitory control in young children have been developed (Petersen et al., 2016). In this study, we used the Head‐toe task from the International Development and Early Learning Assessment (IDELA) (Pisani et al., 2015), which required children to do the opposite of what the tester said, that is, touching their head when instructed to touch their toes and vice versa. The interrater agreement and the test–retest reliability were 0.89 and 0.80, respectively.

#### 1.4.2 Declarative memory tasks

The RACER declarative memory task was developed based on the Paired‐Associate Learning (PAL) paradigm (Hannula et al., 2006). Twelve shapes were randomly grouped into 6 pairs. In the first two blocks, each pair of associated shapes was presented twice. Then, the other RACER tasks were administered, followed by the second two blocks, in which each pair was again presented twice. The second two blocks were presented after a median delay of 11 (10–13) min. In each trial, a target shape appeared at the top of the screen and four shapes across the bottom of the screen. Children were instructed to complete a pair by touching the correct pair‐associated shape from the four options. They continued selecting shapes until they touched the correct pair and were given feedback that they had made the correct choice. The first appearance of each pair was a baseline trial in which the child had the opportunity to learn the associated pair, and subsequent appearances were recall trials. The score on each trial was whether the child correctly completed the pair with their first choice (yes or no). We expected that children in Ghana would show declarative learning by achieving a higher correct proportion on their first choice in the last recall trial than in the baseline trial.

The paper‐based paired‐associate learning task was based on a task developed by Baddeley et al. (1995). In the first part of the test, the tester taught the child novel words for pictures of eight objects, and the child was instructed to point to the correct object as the tester said each one aloud. After a median delay of 7 (6–11) min, the child was given two attempts to point to the correct object representing each word and scored on the number of correct responses. The interrater agreement and test–retest reliability were 0.95 and 0.69.

#### 1.4.3 Procedural memory task

The RACER procedural memory task was developed based on the “Serial Reaction Time” (SRT) paradigm (Nissen & Bullemer, 1987). A total of 175 dots were presented one at a time in one of four locations on the tablet screen. Children were instructed to touch each dot as quickly as possible. If a child was not able to touch a dot within 1000 ms, the dot disappeared, and the trial was recorded as missing. Five blocks were presented in the following order: random‐ordered‐ordered‐random‐ordered. We calculated accuracy as the Euclidean distance from the correct location to the location where the child touched. We expected that children in Ghana would show procedural learning through faster RTs on the final ordered (challenge) block compared to the final random (baseline) block, which is the pattern that has been consistently reported in previous studies (Conway et al., 2019; Lum et al., 2010).

### 1.5 Data processing

The trials with missing responses and RT shorter than 100 ms were excluded from the analyses. Children were excluded from analyses for a given task if they did not respond to at least 50% of the total trials in that task. We calculated the child's height‐for‐age z‐scores (HAZ) according to World Health Organization norms (World Health Organization, 2006).

### 1.6 Statistical analysis

All analyses were prespecified in a Statistical Analysis Plan, and they are available on the Open Science Framework at https://osf.io/fvupk/.

To examine whether our RACER data from children in Ghana replicated previously documented patterns of inhibitory control, declarative memory, and procedural memory (Aim 1), we conducted several mixed‐effects regression analyses, described below. All models included a random effect of a participant on intercept to adjust for repeated trials within participants and prespecified covariates. We used the same regression models, stratified by each child ID, to calculate each child's individual score on each task. All scores were calculated so that higher scores represented better inhibitory control, declarative memory, or procedural memory.

For the Simon task, two linear mixed‐effects regression models were conducted to test whether participants responded significantly more quickly and accurately in the same‐side trials than in the opposite‐side trials. RT and accuracy are likely to be jointly determined; therefore, we controlled accuracy when the performance measure was RT and vice versa. The child's Simon RT score was the coefficient indicating the difference in RT between same‐side and opposite‐side trials, controlling for accuracy. The child's Simon accuracy score was the coefficient indicating the difference in accuracy (horizontal distance from the touch to the target) between same‐side and opposite‐side trials, controlling for RT.

In the PAL task, we used a logistic mixed‐effects regression model to analyze whether the probability of children choosing the correct pair on their first choice was higher in the last recall trial than in the baseline trial, controlling for which shape pair was presented in the item. The child's PAL score was the coefficient indicating the difference in the probability of choosing the correct pair on the first choices between the baseline trial and the last recall trial, controlling for shape pair.

In the SRT task, we applied a linear mixed‐effects regression model to examine whether participants showed a procedural learning effect by responding more quickly in the last ordered block than in the last random block, controlling for accuracy, the stimuli location (one of four locations on the screen), and a binary variable representing whether the dot appeared in the same location as in the previous trial. The child's SRT score was the coefficient indicating the difference in RT between the last ordered block and the last random block, controlling for accuracy, stimuli location, and whether a dot appeared in the same location as the previous trial.

To assess convergent validity and discriminant validity (Aim 2), we examined the Pearson's correlations of participants’ abilities (declarative memory and inhibitory control) measured by the RACER tasks with corresponding and unrelated paper‐based tasks (2a) and child age (2b). To assess the sensitivity to risk factors (Aim 3), we examined the Pearson's coefficients for the correlation of five risk factors (HOME score, maternal education, household asset score, child Hb concentration, and HAZ) with the children's abilities measured by the three RACER tasks, and the correlation of the same risk factors with the paper‐based test scores.

## 2 RESULTS

### 2.1 Participants

We enrolled 966 children at age 4–6 years whose mother or caregiver provided informed consent. We previously reported that the 966 children enrolled in the follow‐up sample were similar to the 354 enrolled in the parent trial but lost to follow‐up in characteristics such as maternal education and household socioeconomic status (Ocansey et al., 2019). Among the 966 participants enrolled, we excluded 22 (2.3%), 24 (2.5%), and 70 (7.2%) participants from the Simon, PAL, and SRT tasks, respectively, due to administration problems, such as child refusal to complete the task and other technical issues. In addition, participants who did not respond to at least 50% of the total trials were excluded from the particular task, which led to another 13(1.3%) participants excluded from the Simon task and 292(32.6%) participants excluded from the SRT task. Children excluded from the PAL and SRT tasks were significantly younger than those included in the study (PAL: *p* < .01; SRT: *p* < .001), and more boys were excluded in the SRT task (*p* < .01) (Table 1). Considering a possible sampling bias due to excluding a large proportion of participants, we conducted the SRT analysis on both the eligible participants (*N* = 604) and all participants who completed the task (*N* = 896). The findings of data analysis were very similar in both samples. We report the results from the eligible participants in this paper, and the results of the full sample are presented in the Supplementary Material.

**TABLE 1:** Comparison of participant characteristics of the included children vs. the excluded children

	Included participants	Excluded participants	
Variable	Mean ± SD [*n*] or % [*n*/total]	Mean ± SD [*n*] or % [*n*/total]	*p* Value
Inhibitory control task	*n* = 931	*n* = 35	
Age (year)	4.92 ± 0.56 [931]	4.84 ± 0.53 [35]	.36
Male (%)	47.53% [442/930]	54.29% [19/35]	.43
Maternal education (year)	7.61 ± 3.53 [931]	6.97 ± 3.76 [35]	.37
Household asset score (×100)^1^	0.50 ± 96.99 [928]	3.62 ± 86.42 [35]	.84
Declarative memory task	*n* = 942	*n* = 24	
Age (year)	4.93 ± 0.56 [942]	4.63 ± 0.41 [24]	<.01
Male (%)	48.14% [453/941]	33.33% [8/24]	.86
Maternal education (year)	7.58 ± 3.57 [942]	7.83 ± 1.97 [24]	.15
Household asset score (×100)^1^	0.27 ± 96.69 [939]	13.8 ± 93.2 [24]	.49
Procedural memory task	*n* = 604	*n* = 362	
Age (year)	5.03 ± 0.55[604]	4.73 ± 0.51[362]	<.001
Male (%)	44.37% [268/604]	54.44% [190/349]	<.01
Maternal education (year)	7.71 ± 3.60 [604]	7.38 ± 3.43 [362]	.15
Household asset score (×100)^1^	–2.83 ± 98.11 [602]	6.36 ± 93.85 [361]	.15

^1^ Proxy indicator for household socioeconomic status constructed for each household based on ownership of a set of assets (radio, television, etc.), lighting source, drinking water supply, sanitation facilities, and flooring materials. Household ownership of this set of assets is combined into an index (with a mean of zero and standard deviation of one) using principal components analysis. A higher value represents higher socioeconomic status.

### 2.2 Aim 1: Replication of previous patterns of effects

#### 2.2.1 RACER Simon task

**FIGURE 1:**
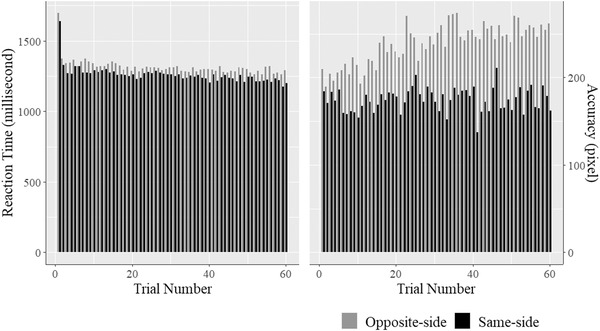
The average reaction time and accuracy in the opposite‐side trials versus the same‐side trials

Figure 1 shows the average RT and accuracy across participants by trial in opposite‐side trials and same‐side trials. As expected, the results showed significant coefficients (*γ*) for trial type on both RT (*γ* = −56.77, *SE* = 3.46, *p* < .001), indicating that children responded on average 57 ms faster on same‐side trials (*M* = 1264.97, *SD* = 7.01) than opposite‐side trials (*M* = 1321.74, *SD* = 7.01), and accuracy (*γ* = −65.90, *SE* = 2.67, *p* < .001), indicating that children responded on average 66 pixels closer to the target on same‐side trials (*M* = 214.90, *SD* = 6.10) compared to the opposite‐side trials (*M* = 280.80, *SD* = 6.22).

#### 2.2.2 RACER PAL task

**FIGURE 2:**
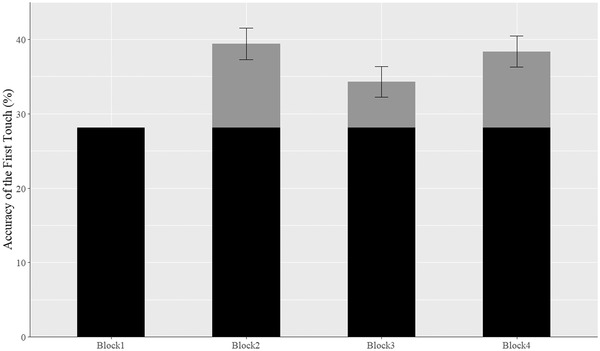
The average percentage of the correct first touches across eligible participants (*N* = 942) for the Blocks. *Note*: The *y*‐axis represents the mean accuracy across participants, with accuracy defined as the percentage of trials (pair of shapes) correctly identified in the first response out of 6 trials presented in each block. The *x*‐axis represents each of the four blocks in the task. For Blocks 2, 3, and 4, the blue bar shows the mean accuracy on block 1 and the black bar shows the increase in mean accuracy on that subsequent block, which demonstrates learning and memory. The error bars represent the 95% confidence interval of the difference from baseline (accuracy on Block 1)

As expected, the result showed a significant positive coefficient for the last recall block compared to the first block (*γ* = 0.463, *SE* = 0.05, *p* < .001), which indicated that the odds of selecting the correct shape on the first choice in the last block were 1.589 ( = *e* ^0.463^) times greater than the odds in the baseline block. The differences between the first and the last block are shown in Figure 2, in which the average probability increased more than 10 percentage points in the last block.

#### 2.2.3 RACER SRT task

**FIGURE 3:**
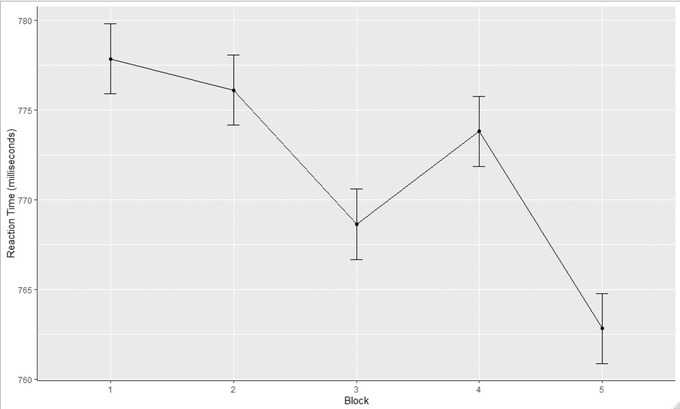
Reaction time across all eligible participants (*N* = 604) for the five blocks. *Note*: The *x*‐axis represents each of the five blocks in the task. The *y*‐axis represents the mean response time across participants for each block. The error bars represent the 95% confidence interval

Figure 3 shows the average RT across all eligible participants in each of the five blocks. As we expected, the linear mixed‐effects regression model showed a significant coefficient for block 5 versus block 4 (*γ* = 13.38, *SE* = 1.32, *p* < .001), which indicated that participants made responses on average 13.38 ms faster in the last ordered block (*M* = 756.02, *SD* = 2.19) than the last random block (*M* = 769.39, *SD* = 2.19).

### 2.3 Aim 2: Convergent validity, discriminant validity, and increase in scores with age

#### 2.3.1 Validity

No RACER score was significantly correlated with any paper‐based task score. The Pearson correlation between the RACER PAL task and the paper‐based PAL task was *r* (639) = −.06, *p* = .14. Neither of the inhibitory control scores was significantly correlated with the Head‐toe task score (RT: *r* (927) = −.04, *p* = .24; accuracy: *r* (927) = −.06, *p* = .10). All other correlations also showed nonsignificant results (RACER PAL vs. Head‐toe: *r* (639) = −.002, *p* = .96; inhibition RT vs. paper and pencil PAL: *r* (927) = −.06, *p* = .08; inhibition accuracy vs. paper and pencil PAL: *r* (927) = −.06, *p* = .09).

#### 2.3.2 Increase in scores with age

RACER Simon accuracy score was significantly correlated to child age (*r* (929) = .09, *p* = .007), which indicated that older children showed better inhibitory control abilities than younger children in the sample. However, neither children's inhibitory control abilities measured by RACER Simon RT (*r* (929) = .04, *p* = .24) nor their declarative memory scores measured by RACER PAL (*r* (940) = −.05, *p* = .12) correlated with their age.

### 2.4 Aim 3: RACER and risk factors

None of the RACER tasks’ scores significantly correlated to any environmental risk factor (Table 2). In contrast, the paper‐based inhibitory control head‐toe task showed significant correlations with HOME inventory, maternal education, and HAZ. The paper‐based declarative memory PAL scores were significantly correlated with HOME inventory scores and maternal education.

**TABLE 2:** Correlation between risk factors and cognitive abilities measured by RACER and paper‐based tasks

	Inhibitory control scores			Declarative memory scores		Procedural memory scores
Risk factor	Paper‐based head‐toe task	RACER by reaction time	RACER by accuracy	Paper‐based PAL task	RACER PAL task	RACER SRT task
Home inventory	*r*(616) = .11**	*r*(893) = –.06	*r*(893) = .05	*r*(919) = .18***	*r*(618) = –.02	*r*(584) = .04
Household SES	*r*(638) = .01	*r*(926) = .01	*r*(926) = .05	*r*(953) = –.02	*r*(640) = –.04	*r*(600) = –.03
Maternal education	*r*(639) = 0.12**	*r*(929) = .01	*r*(929) = .02	*r*(956) = .11***	*r*(641) = .04	*r*(602) = .01
Hemoglobin concentration	*r*(574) = .04	*r*(829) = .03	*r*(829) = –.04	*r*(854) = .02	*r*(576) = .06	*r*(534) = –.05
Length‐for‐age *Z*	*r*(636) = .07	*r*(924) = .03	*r*(924) = .05	*r*(951) = .07*	*r*(638) = –.03	*r*(600) = .06

* *p* < .05.

** *p* < .01.

*** *p* < .001.

PAL: paired‐associated learning; RACER: rapid assessment of cognitive and emotional regulation; SES: socioeconomic status; SRT: serial reaction time.

## 3 DISCUSSION

The development of easily administered, cost‐ and time‐efficient cognitive assessment tools for use on a large scale in LMICs is urgently needed. Among children aged 7–18 years, the RACER has previously been shown to be feasible and valid to measure children's inhibitory control and memory abilities in LMICs. Our investigation in preschool children aged 4–6 years in Ghana replicated expected patterns of responses showing inhibitory control and declarative and procedural learning (Aim 1). However, our findings did not support our hypotheses of acceptable convergent validity between RACER and paper‐based tasks (Aim 2a). In addition, the RACER tasks did not exhibit more sensitivity to the risk factors commonly experienced by preschool children in LMICs than the paper‐based tasks (Aim 3).

The high percentage of children who were able to complete the tasks shows that RACER is an easy‐to‐use assessment for measuring child cognitive development in an LMIC setting. We successfully administered two RACER tasks (Simon task and PAL) to 966 children aged 4–6 years in Ghana. The percentage of children from whom we could not obtain any data on the RACER Simon, PAL, and SRT tasks was low: 2.3%, 2.5%, and 7.2%, respectively. This indicates that administration of these tasks in a tablet‐based format was tolerated well by these young children, despite the likelihood that they may have had less experience with tablets than their peers in HICs. This also supports the previous findings from studies using the RACER tasks in Lebanon and Niger (Ford et al., 2019). However, many of the children (32.6%) were excluded from the data analysis on the SRT task because they did not respond to at least 50% of the total trials in that task, and excluded children were significantly younger than those who were able to complete the task. While 81% of 5–6 year olds were able to complete at least half the trials, only 57% of 4‐year‐olds were able to complete at least half of trials. This suggests that this task should not be used for most children as young as 4 years and also may not be appropriate for some 5‐ to 6‐year‐olds.

Among children who provided analyzable data, the pattern of results that we found among young children in Ghana replicated previously documented effects on similar tasks assessing inhibitory control, declarative memory, and procedural memory among children the same age in HICs. Semrud‐Clikeman et al. (2017) highlighted that measures developed in HICs were not necessarily appropriate for measuring the same underlying constructs when administered in different cultures and that they need to be investigated. The findings of this study suggest that tablet‐based tasks may be promising tools for adapting widely used cognitive tests originating in HICs to LMIC settings.

Although we found expected performance patterns, we did not find that the RACER tasks showed convergent validity or sensitivity to environmental risks. One possible reason for the unexpected findings on Aims 2 and 3 is that many children showed a “floor effect.” Although almost all children were able to perform the tasks, many of their task scores were not in the expected direction (PAL: 182; Simon‐RT: 342; Simon‐Accuracy: 364; SRT: 383 children). Among the three tasks, the SRT task seemed to be the most challenging. Almost one‐third (32.6%) of participants did not make a response for more than 50% of the trials within the 1000 ms time window and were, therefore, excluded from the analysis. Although RACER and other tablet‐based tasks have been shown to be valid for children aged 6 years and older, this study suggests that further improvement or adjustment may be needed for children younger than 6 years of age.

Another possible reason for the unexpected findings is the relatively small number of trials in each RACER task. Although RACER was developed based on widely used test paradigms, the administration duration and trial numbers were tailored for use in large field trials in LMICs, and were, therefore, designed to maximize ease of administration and time efficiency. For instance, previous studies (Conway et al., 2019; Lum et al., 2010) assessed procedural memory using an SRT task and used at least 60 trials for each block, while each block in the RACER SRT task consisted of 35 trials. The limited number of trials may reduce the power to identify individual differences on those tasks, especially for younger children.

Compared to paper‐based tests or experiments using nontouchscreen devices to collect participant responses, such as keyboards or response boxes, tablet‐based tasks have the advantage of capturing the precise location that a participant touches. To assess inhibitory control using the RACER task, we used two performance measures, RT in milliseconds and accuracy in pixels. The analysis revealed that inhibitory control ability as measured by accuracy was significantly related to child age, as expected, while inhibitory control ability based on RT was not. As shown in Figure 1, there was a larger difference in children's performance in between opposite‐side trials and same‐side trials in accuracy than in RT, which suggests that accuracy was a better indicator of children's inhibitory control abilities. This study has several strengths. This is one of the first studies to explore the feasibility and validity of a tablet‐based tool in measuring fundamental cognitive abilities in young children in an LMIC. Besides examining the validity, we also measured the sensitivity of the RACER tasks to the common risk factors that young children experience in LMICs. In addition, we collected behavioral measurement data and risk factor data from a large sample of children that was representative of the full trial sample in a relatively cost and time‐efficient way, which allowed us to evaluate multiple psychometric properties of the RACER tasks. However, this study also has several limitations. Children in the sample were in a limited age range (4–6 years). Many of the children (32.6%) were excluded from the data analysis on the SRT task. We were not able to test our speculation that the lack of sensitivity of the test scores may be due to a tendency toward the “floor effect” for younger children.

This study did not provide evidence to support that RACER is a valid tool to capture individual cognitive differences among children at 4–6 years of age in Ghana. In the future, examining the psychometric properties of RACER with older children may provide a clearer picture of how well RACER performs versus paper‐based tasks. To maximize ease of administration and time efficiency for use in large field trials in LMICs, we reduced the trial numbers and limited the response time window for each trial, which may have led to a “floor effect” for some children. Future improvements to the RACER tasks could include extending the RT window, reducing the complexity of the learning stimuli, shortening the stimuli sequence, and providing additional learning trials and real‐time feedback during the training session.

## CONFLICT OF INTEREST

The authors report no conflict of interest.

### PEER REVIEW

The peer review history for this article is available at https://publons.com/publon/10.1002/brb3.2749

## Supporting information

Supplementary Material

## Data Availability

The data that support the findings of this study are available on request from the corresponding author. The data are not publicly available due to privacy or ethical restrictions.
