# Supplementary material for: Evaluation of a tablet‐based assessment tool for measuring cognition among children 4–6 years of age in Ghana
Source: Brain Behav. 2022 Sep 9;12(10):e2749. doi: 10.1002/brb3.2749 (PMC9575601; doi:10.1002/brb3.2749)
Supplement: Supplementary file 1 — Supplementary Material [file BRB3-12-e2749-s001.docx]

Supplementary Material

Considering a possible sampling bias due to excluding a large proportion of participants in the RACER SRT task, we conducted data analysis on all participants who completed the task (*N* = 896), as well as the eligible participants (*N* = 603). The analysis results of all participants in the SRT task are presented in this supplementary material.

**Aim 1. Replication of Previous Patterns of Effects**

Figure 1 shows the average RT across all participants in each of the five blocks. The response pattern of all participants was similar as what we expected and those of the eligible participants (*N* = 603). The linear mixed-effects regression model showed a significant coefficient for block 5 versus block 4 (*b* = 10.99, *SE* = 1.26, *p* < .001), which indicated that participants made responses on average 10.99 millisecond faster in the last ordered block (*M* = 762.82, *SE* = 1.96) than the last random block (*M* =773.81, *SE* =1.96).

*Figure1. Reaction Time across All Participants (N = 896)*


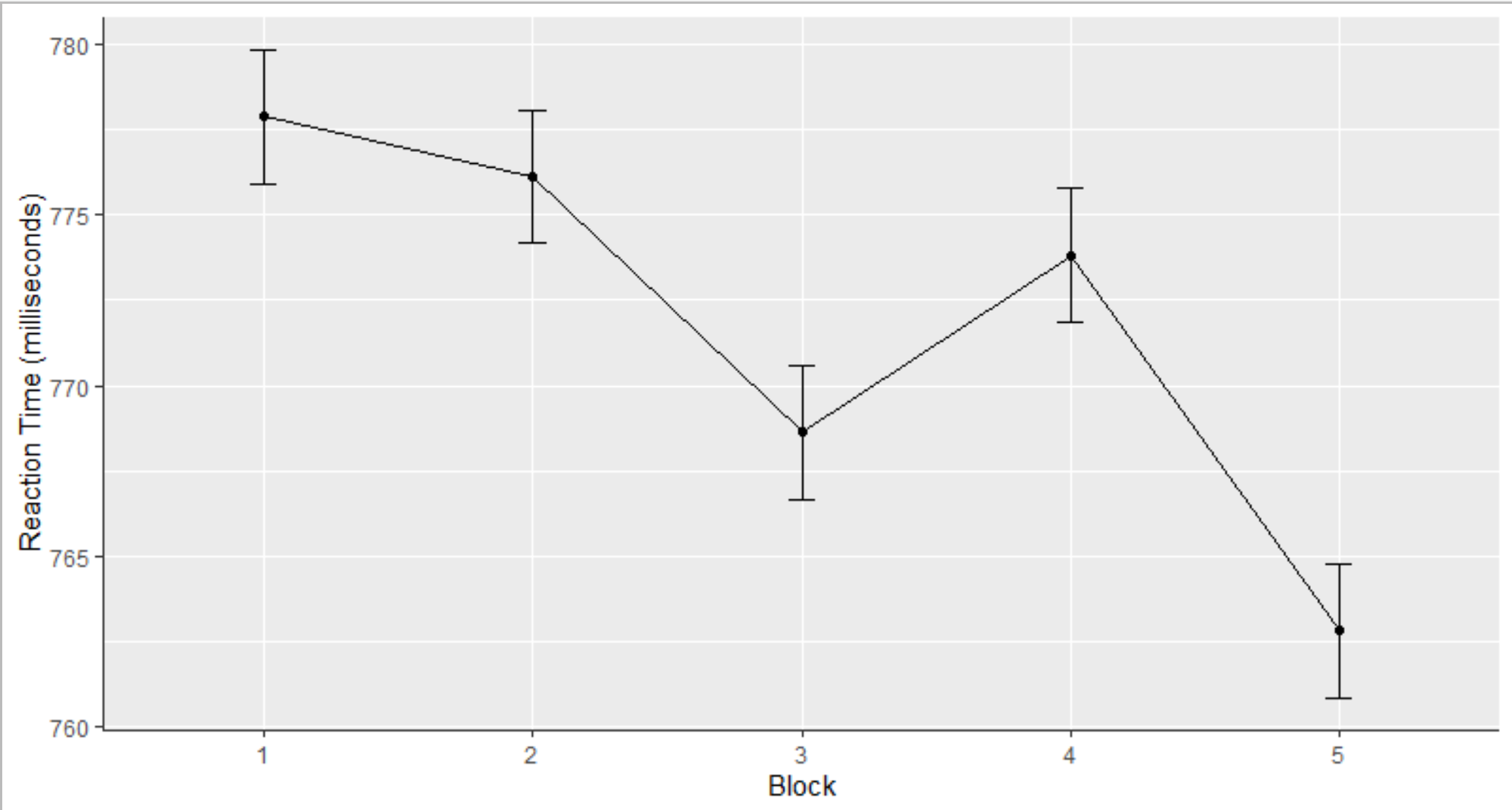


**Aim 3. RACER and Risk Factors**

The results of Pearson’s correlation on all participants shows that none of the SRT scores significantly correlated to any environmental risk factors (Table 1). This finding is same as the one from the data analysis on the eligible participants (*N* = 604).

**Table 1**

*Correlation between Risk Factors and the SRT Scores Measured by RACER*

|  | Home Inventory | SES | Maternal Education | Hemoglobin Concentration | Length-for-age Z |
| --- | --- | --- | --- | --- | --- |
| SRT Scores | *r*(818) = 0.03 | *r*(846) = -0.03 | *r*(849) = 0.01 | *r*(752) = -0.05 | *r*(845) = 0.03 |
